# Low mutation rate in the *TTN* gene in paediatric patients with dilated cardiomyopathy – a pilot study

**DOI:** 10.1038/s41598-019-52911-1

**Published:** 2019-11-11

**Authors:** Elena Zaklyazminskaya, Vadim Mikhailov, Anna Bukaeva, Natalia Kotlukova, Inna Povolotskaya, Vladimir Kaimonov, Anna Dombrovskaya, Sergey Dzemeshkevich

**Affiliations:** 1 Petrovsky National Research Center of Surgery, 2, Abricosovsky side-street, 119991 Moscow, Russia; 2 Pirogov Russian National Research Medical University, Moscow, Russia; 3 Bashlyaeva Pediatric City Hospital, Moscow, Russia; 4 Centre of Genetics and Reproductive Medicine “Genetico”, Moscow, Russian Federation

**Keywords:** Disease genetics, Cardiovascular genetics

## Abstract

Idiopathic dilated cardiomyopathy (DCM) is a common cardiomyopathy with the prevalence of 1:250, and at least one-third of all the cases are inherited. Mutations in the *TTN* gene are considered as the most frequent cause of inherited DCM and cover 10–30% of the cases. The studies were mainly focused on the adult or mixed age group of patients with DCM. The mutation rate in the *TTN* gene, the characteristics of manifestations and their prognostic significance in childhood have not been studied. To determine *TTN* mutation rate in children with DCM and the relevance of including this gene in the DNA diagnostic protocol for paediatric DCM, complete clinical and instrumental examination of 36 DCM patients (up to 18 years) with the manifestation of the disease was conducted in specialised cardiology centres. Molecular genetic testing included sequencing of coding and adjacent regulatory regions of the major cardiac *TTN* isoform N2BA using IonTorrent ™ semiconductor sequencing (for 25 isolated cases) and trio whole exome sequencing (trio WES)on the Illumina platform (for 11 family cases). Our pilot group included 36 probands with *DCM* diagnosis first established on the basis of the generally accepted criteria at the age of 5 days to 18 years(average age: 6.5 years). The sex ratio (M:F) was 23: 8. There were 25 sporadic DCM cases and 11 cases of familial DCM (at least one of the parents and/or siblings were also diagnosed with DCM). The only likely pathogenic truncating variant p.Arg33703*in the *TTN* gene (TTNtv) was found in a 16-year-oldmale proband out of 36 (3%). Apparently, *TTN*-dependent forms of DCMs manifest later at a young (but older than 18 years) or more mature age, and *TTN* gene cannot be considered as the first-line genetic testing for DCM in the paediatric group, despite several studies have reported a generally high mutation rate in this gene with DCM. Further research is needed to compare the representation of mutations in the *TTN* gene in different age groups of DCM patients.

## Introduction

Dilated cardiomyopathy (DCM) is one of the common hereditary diseases, with the prevalence of 1: 250 as estimated by Hershberger *et al*.^1^. The annual incidence of this disease in children was 0.57 cases per 100 000 per year overall in the USA alone; DCM detection rate in other countries was also similar^2^.

DCM may develop under the influence of genetic (primary) or non-genetic (secondary) factors, the frequency of primary forms in the paediatric group of patients reaches 70%^2,3^. DCM in children is characterised by rapid progression and high mortality^4^. For paediatric patients, the five-year probability of death or heart transplantation is 46%^2^. As little is known about the causes, including genetic ones, of DCM in children, the treatment protocols largely represent adapted protocols for the treatment of adult patients, and their effectiveness is limited. For the radical management of progressive heart failure in children and adults, orthotopic heart transplantation (not available in all countries) and mechanical circulatory support devices^5^ are currently used. It is therefore not surprising that during the medical genetic counselling for this severe and steadily progressive disease, the parents have important questions about verifying the genetic diagnosis, knowing the risk of giving birth to a child with this disease, and the prospects of pre-symptomatic (including prenatal) diagnosis and pre-implantation genetic screening (PGS). However, assisted reproductive treatment can be offered to a family only if the pathogenic mutation has been identified.

Among all primary cardiomyopathies, the approaches to DCM DNA testing are the least developed. In 2011, the expert consensus recommendations for the genetic diagnosis of cardiomyopathies and channelopathies stated that none of the known genes was responsible for >5% of primary DCM cases, there was no protocol for priority DNA testing^6^. In 2016, ESC Guidelines for the diagnosis and treatment of acute and chronic heart failure pointed out the *TTN* gene as the most frequent genetic cause of primary DCM^7^. First, this situation is associated with exceptional genetic diversity of DCM. At least 50 genes have a significant role in DCM development; however, the contribution of each gene to the structure of the disease has not been studied enough^8^. A small but consistently detectable proportion of mutations was shown only for mutations in the *SCN5A* and *LMNA* genes for a particular clinical DCM variant accompanied by atrioventricular (AV) conduction impairment (5–10% each) and the *TTN* gene^6^. However, the genetic structure of this disease is poorly studied, and the mutations in each gene are not identified because of the large variety of genes and the absence of frequent mutations. Only with the development of new generation sequencing methods (NGS), large-scale genetic studies of this disease were conducted. The only gene in which mutations account a significant proportion of DCM probands - the *TTN* gene encoding titin sarcomeric protein.

Before the introduction of NGS methods, the *TTN* gene was poorly studied because of its large size^8^. But several studies have shown that mutations in this gene are the leading cause of DCM^8–12^. According to various estimates, from 10 to 30% of DCM cases are caused by mutations in the *TTN* gene^2,11^. The first estimates of the prognostic value of detecting mutations in this gene were performed on several patients with identified mutations. The survival and long-term prognosis of DCM patients may depend on the genetic form of the disease^13^. In addition, *TTN* gene mutations have incomplete penetrance, and in DCM patients, a titin mutation is often aggravated by pathogenic variants in other DCM-related genes^14^.

Previous researches were mainly focused on the adult or mixed age group of patients with DCM^15^. Only a few studies investigated the spectrum of mutations in the paediatric and adolescent groups of patients^16^. The *TTN* mutation rate, the characteristics of manifestations and their prognostic significance in childhood were not thoroughly studied.

## Aim

The aim of the study was to determine the *TTN* mutation rate in paediatric patients with DCM and to assess the relevance of the inclusion of this gene into the DNA diagnostics protocol.

## Materials and Methods

Complete clinical and instrumental examination of the patients were performed in accordance with diagnostic protocol at the specialised cardiology and cardiac surgery centres and departments where children with DCM were diagnosed and/or observed. Genetic counselling and molecular genetic research were performed in accordance with the principles of the Declaration of Helsinki, informed consent for genetic testing was given by the parents or guardians of the minor patients. The informed consent included DNA diagnostics and using the results of the study for publishing. The results do not violate the confidentiality of patients and could not be used to identify them.

Molecular genetic analysis was conducted on the DNA extracted from the venous blood samples or from paraffin blocks (in three cases, DNA testing was performed post- mortem) with standard reagent kits. For 25 probands (sporadic cases, the only patient in family), sequencing of coding and adjacent regulatory regions of major cardiac *TTN* isoform N2BA using IonTorrent ™ semiconductor sequencing was performed.

For 11 probands (family cases) and their parents, trio whole exome sequencing (trio WES) on the Illumina platform was performed. Capillary Sanger re-sequencing was performed for the fragments with low coverage and for the confirmation of the identified genetic variants.

All genetic variants annotated by original software were analysed with open bioinformatics tools.

We used PolyPhen, Mutation Taster, SIFT, FATHMM, HSF, Cardio Classifier and Atlas of Cardiac Genetic Variation Database (https://www.cardiodb.org)^17–22^.

For primary analysis of sequencing results we used IGV (Interactive Genomic Viewer), and variant tables were created by IonRep software^23^. Every variant found was proof-checked in online genome browser (UCSC browser) and variant databases (dnSNP, gnomAD)^24–26^. Genetic variant with the frequency >1% was not considered as potentially pathogenic. Human Splicing Finder and NetGene2 were used to assess the potential impact on splicing^27,28^.

### Ethical approval

This study was performed in accordance with the 1964 Helsinki declaration, its later amendments and local ethics committee.

### Informed consent

Written informed consent was obtained from all individual participants included in the study.

## Results and Discussion

The study group included 36 probands diagnosed with *DCM* first established on the basis of the generally accepted criteria at the age of 5 days to 18 years (in one case, DCM with the left ventricular non-compaction was diagnosed prenatally at the period of 30 weeks of gestation); the average age was 6.5 years. The sex ratio (M:F) was 23: 8. There were 25 sporadic DCM cases and 11 cases of familial DCM (at least one of the parents and/or siblings was also diagnosed with DCM). Clinical phenotype of patients at the time of the first consultation is summarized in the Table 1 (supplementary).

On the basis of the published reports of a significant (up to 30%) frequency of pathogenic genetic variants in the *TTN* gene^3,8^ that lead to shortened protein isoform development, we were expected to detect at least 6–8 mutations in the examined group. However, the only one truncating variant p.Arg33703* was found in a cohort of patients with paediatric DCM.

**Figure 1:**
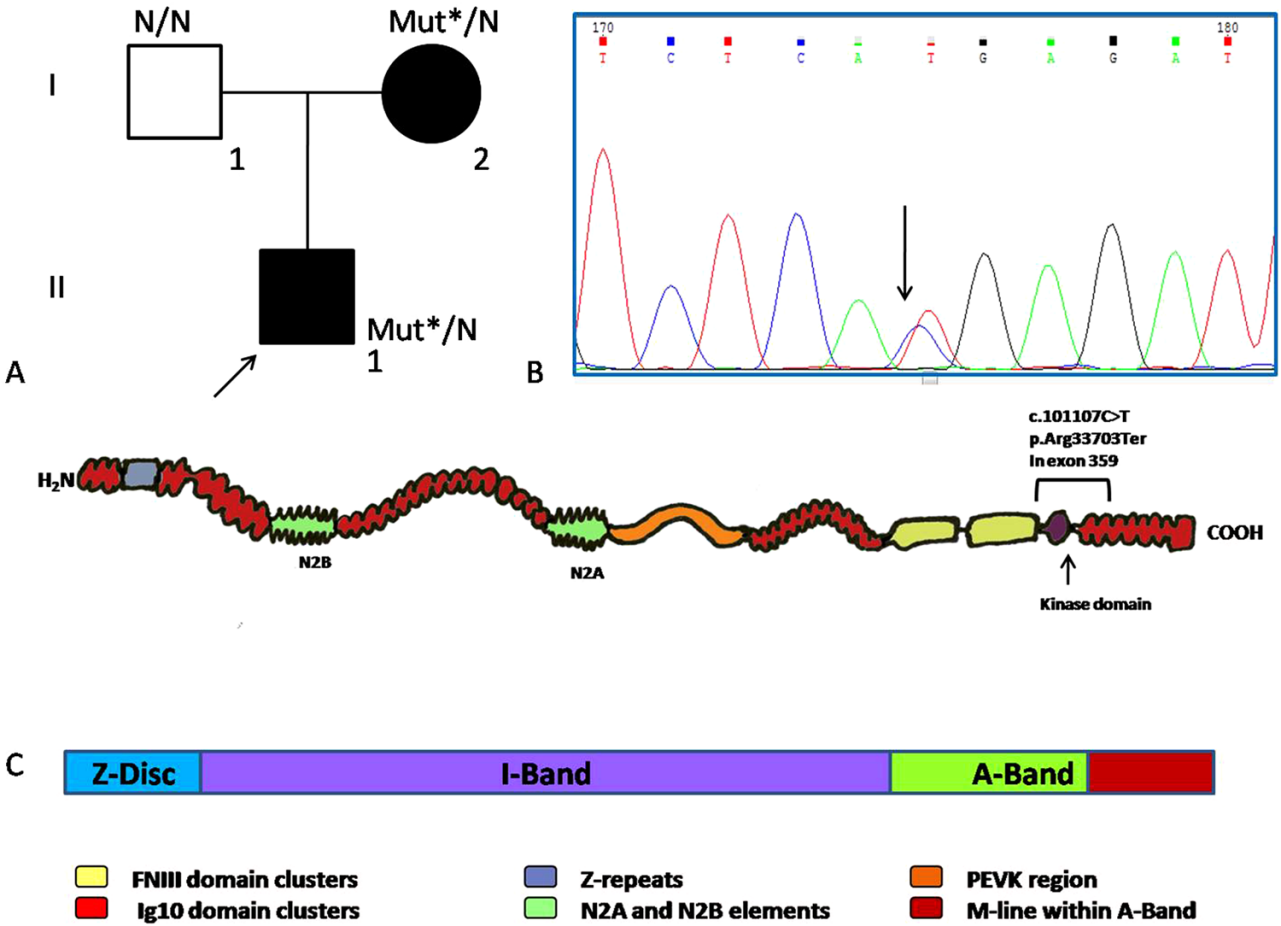
(**A**) Stop-gain variant carrier family tree. I.2–41 y.o., DCM, Heart failure (HF) NYH III-IV; II.1–16 y.o., DCM, HF NYH II; Mut* - c.101107 C > T (p.Arg33703Ter). (**B**) Fragment of the Sanger sequencing chromatogram with heterozygous C > T substitution. (**C**) Titin protein scheme and its alignment with the regions of the sarcomere. p.Arg33703Ter location is shown.

The carrier of this variant was diagnosed with DCM at 16 years. The main complaint was palpitation caused by PVC. The left ventricle (LV) was slightly enlarged (EDD (end diastolic volume) LV 57 mm) with decreased ejection fraction (EF LV 45%), but the tolerance to the physical exercise was preserved. This variant was also found in the proband’s mother (41 y.o.) with syncope, DCM, heart failure (NYHA IV), pulmonary hypertension, supraventricular and ventricular arrhythmia (Fig. 1A).

This is a heterozygous variant ENST00000589042.1:c.101107 C > T p.Arg33703* located at exon 359 in the IC isoform (exon 308 for N2BA isoform) (Fig. 1B,C). This variant is extremely rare (MAF 0,00001797 in the gnomAD) and is registered in the dbSNP database (rs766265889); however, no clinical data are available. Exon 359 translates within the M-disc of the sarcomere and encodes 9 domains (Ig-like 142–148, fibronectin-type III 132 and protein kinase domains). The exon is asymmetric, and its PSI (Proportion Spliced In) is 100% in DCM patients. rs766265889 has two entries in ClinVar database (one of which is our submission), and there are a number of other truncating variants mapped in exon 359 with p.Asp33700fs as the most closely located (https://www.cardiodb.org/titin/titin_exon.php?id=359). The location of the exon within M-disc, its large size and multi-domain composition, asymmetry and PSI allows us to treat p.Arg33703Ter as a likely pathogenic finding, but the exact role of the p.Arg33703Ter has to be elucidated.

Generally, deletions leading to a frameshift result in a premature termination codon and are considered pathogenic by default. But the clinical interpretation of genetic variants found in the *TTN* gene is very complex because of the enormous size of the protein and the objective difficulty of performing functional analysis of mutations, and also the high frequency of occurrence of premature termination codons and mutation splicing in healthy population, which reaches 2–3%^1,8^. It has been shown that the clinical effect of mutations, even leading to premature termination codons and changes in splicing sites, strongly depends on which domain of the protein they affect. Currently, the focus is on genetic variants with a more unambiguous effect on the protein – splicing mutations and nonsense mutations.

The bioinformatics research showed that the pathogenicity of a variant in the *TTN* gene depends on how widely the exon with the mutation is represented in all isoforms of the protein and which domain it affects^15^. Pathogenic mutations are grouped by gene segments corresponding to specific structures in the protein molecule in the composition of the sarcomere^12^. A significant portion of premature termination codons qualified as pathogenic are localised in the C-terminal domain of the cardiac-specific isoforms and translating within A-disc or M-disc of the sarcomere^12,29^.

The results of this study were somewhat unexpected: a single mutation in the *TTN* gene was detected in patients with DCM diagnosed before the age of 18 years. These data can only partly be explained by the limited size of the sample or ethnic characteristics of the Russian group of patients. Our results are consistent with those of D. Fatkin *et al*., where only a small number of TTNtv are found in the group of 82 children with DCM including one *de novo* mutation^16^.

The structure of the genetic causes of DCM is different at different ages. Apparently, *TTN*-dependent forms of DCM manifest later, at a young (but over 18 years) or at a more mature age. Data on the genotype–phenotype correlation with DCM are insufficient. Studies on the mid-term survival of DCM patients showed that mutations in the *TTN* gene are not associated with deterioration in the prognosis compared with other patients with DCM^15^. Data have shown that patients with *TTN*-mediated DCM have a better prognosis compared with *LMNA*-dependent cardiomyopathy considering the rate of heart failure progression and the high risk of life-threatening ventricular arrhythmias^28,30,31^. However, in all the cases, the discussion was about the examined groups of patients with the average age of clinical manifestation of DCM at 40–50 years^13,16,30^ which, compared with the paediatric group, is certainly a better prognosis.

## Conclusion

The absence of pathogenic substitutions in patients with DCM manifestation at an age less than 18 years indicates the possibility of the existence of different genetic causes of DCM in different age groups. In addition, this suggests a more favourable course of *TTN*-mediated forms of DCM compared to childhood forms of the disease. The pathogenic role of the *TTN* mutations should be assessed with caution. From the results of this study, the *TTN* gene cannot be considered as a *first*-*line* genetic test for DCM in the paediatric group, despite several studies have reported a generally high mutation rate in this gene with DCM. Further research is needed to compare the representation of *TTN* mutations in different age groups of patients with DCM.

## Supplementary information

Supplementary material
