# Supplementary material for: Low mutation rate in the TTN gene in paediatric patients with dilated cardiomyopathy – a pilot study
Source: Sci Rep. 2019 Nov 11;9:16409. doi: 10.1038/s41598-019-52911-1 (PMC6848193; doi:10.1038/s41598-019-52911-1)
Supplement: Supplementary file 1 — Supplementary material [file 41598_2019_52911_MOESM1_ESM.docx]

**Low mutation rate in the *TTN* gene in paediatric patients with dilated cardiomyopathy**

– **a pilot study**

Elena Zaklyazminskaya^1,2^, Vadim Mikhailov^1^, Anna Bukaeva^1^, Natalia Kotlukova^2,3^, Inna Povolotskaya^4^, Vladimir Kaimonov^4^, Anna Dombrovskaya^1^, Sergey Dzemeshkevich^1^

^1^ – Petrovsky National Research Center of Surgery, 2, Abricosovsky side-street, 119991, Moscow, Russia

^2^ – Pirogov Russian National Research Medical University, Moscow, Russia

^3^ – Bashlyaeva Pediatric City Hospital, Moscow, Russia

^4^ – Centre of Genetics and Reproductive Medicine “Genetico”, Moscow, Russian Federation

***Key words:*** *dilated cardiomyopathy, TTN, paediatric cardiomyopathy, DNA diagnostics, truncating mutations.*

**Corresponding author:**

Vadim Mikhailov

Researcher of the Medical Genetics Laboratory

^1^Petrovsky National Research Center of Surgery

2, Abricosovsky side-street, 119991, Moscow, Russia

+7 499 2485495

Supplementary material

Table 1. Clinical characteristics of pediatric DCM patients at the first consultation

| **Patient's code** | **gender** | **age** | **Family/ Sporadic** | **NYHA class** | **arrhythmia/ conduction defects** | **skeletal muscle involvement** | **other extra-cardiac traits** |  |
| --- | --- | --- | --- | --- | --- | --- | --- | --- |
| pDCM1 | m | 16 years | family | class I-II | no changes | no | no |  |
| pDCM2 | m | 16 years | family | class IV | Sinus arrhythmia, atrial conduction delay, PVC, VT | progressive muscular weakness | bilateral ptosis, hypomimic face, horseshoe kidney | PVC premature ventricula beats; VT ventricular tachycardia |
| pDCM3 | m | 17 years | sporadic | class I-II | sinus bradicardia, SAB, ventricular conduction defects | no | arterial hypertension, horseshoe kidney | SAB sinoatrial block |
| pDCM4 | m | 1 year 7 months | sporadic | class II | no changes | no | no |  |
| pDCM5 | m | 1 year | sporadic | class II | no changes | no | no |  |
| pDCM6 | m | 5 months | sporadic | class II | no changes | no | no |  |
| pDCM7 | f | at birth | family | class II-III | no changes | no | no |  |
| pDCM8 | f | 5 years | family | class II | no changes | no | no |  |
| pDCM9 | m | 4 years | family | class II | no changes | no | no |  |
| pDCM10 | m | 6 months | family | class III | no changes | no | no |  |
| pDCM11 | m | 16 years | sporadic | class I-II | no changes | no | no |  |
| pDCM12 | f | 16 days | sporadic | class II | paroxysmal sinus tachicardia | no | nephropathy, pulmonary hypertension |  |
| pDCM13 | m | 5 days | sporadic | class II-III | no changes | no | no |  |
| pDCM14 | m | 17 years | sporadic | class II | paroxysmal sinus tachicardia, supraventricular extrasystoles, PVC | no | no |  |
| pDCM15 | m | 8 years | sporadic | class II-III | sinus bradicardia, AVB (II) | no | obesity | AVB - atrioventricular block |
| pDCM16 | m | 5 months | sporadic | class II | paroxysmal VT, supraventricular extrasystoles, ventricular conduction defects | muscular weakness | no |  |
| pDCM17 | m | 3 years | sporadic | class II | AVB (I) | congenital myopathy, "dropped head" syndrome | adenoiditis, eustachitis, cryptorchidism |  |
| pDCM18 | m | 2 years 10 months | family | class I-II | no changes | no | no |  |
| pDCM19 | f | 5 days | sporadic | class III | no changes | muscle dystonia | no |  |
| pDCM20 | m | 3 months | sporadic | class II-III | sinus bradicardia, PVC | no | no |  |
| pDCM21 | f | 16 years | sporadic | class IV | sinus tachicardia, PVC, ventricular conduction defects | no | pulmonary hypertension, residual neural damage, myopia, secondary glaucoma of left eye |  |
| pDCM22 | f | 7 months | sporadic | class I-II | no changes | no | no |  |
| pDCM23 | m | 3 years | sporadic | class II-III | sinus arrhythmia, ventricular conduction defects | no | drop-head syndrome, muscular weakness |  |
| pDCM24 | m | 1 year 3 months | sporadic | class III | brady/tachicardia; ARVC, AVB (I) | no | chronic rhinitis, moderate myopia, frequent skin infection | ARVC arrhythmogenic right ventricular cardiomyopathy |
| pDCM25 | m | 17 years | family | class II | no changes | no | no |  |
| pDCM26 | f | 1 month | sporadic | class II-III | no changes | no | no |  |
| pDCM27 | f | 2 years 10 months | sporadic | class I-II | no changes | no | no |  |
| pDCM28 | f | at birth | family | class III-IV | arrythmia | no | cerebral coma, acute respiratory failure |  |
| pDCM29 | m | 8 months | family | class II-III | no changes | no | oro-facial cleft |  |
| pDCM30 | f | 9 months | sporadic | class II | chronic supraventricular tachicardia, atrial conduction delay, repolarization impairment | no | no |  |
| pDCM31 | f | 5 months | sporadic | class III | sinus tachycardia, AVB (I) | no | acute CMV infection |  |
| pDCM32 | m | 13 years | family | class II | no changes | no | no |  |
| pDCM33 | f | 2 years | sporadic | class II | PVC | no |  |  |
| pDCM34 | m | 16 years | sporadic | class II-III | ventricular conduction defects | no | no |  |
| pDCM35 | f | 17 years | sporadic | class III-IV | "tachy brady syndrome", ventricular conduction defects | no | no |  |
| pDCM36 | f | n/a | sporadic | class II-III | no changes | no | no |  |
